# Sufentanil inhibits the proliferation and epithelial mesenchymal transition of lung cancer cells through Wnt/beta-catenin signaling pathway

**DOI:** 10.1080/21655979.2022.2066045

**Published:** 2022-04-27

**Authors:** Minghan Guan, Yifeng Huang, Xiaowen Lin

**Affiliations:** a Department of Anesthesiology, Benxi Central Hospital, Mingshan District, Benxi, Liaoning, China; b Department of Anesthesiology, Chongqing Beibu Maternity Hospital, Chongqing, China; c Department of Pain Management, Shandong Provincial Hospital Affiliated to Shandong First Medical University, Jinan, Shandong, China

**Keywords:** Sufentanil, lung cancer, wnt/β-catenin, epithelial mesenchymal transition

## Abstract

Lung cancer is the most common malignancy and leading cause of cancer-related death. Sufentanil is a commonly used opioid anesthetic in clinics. This study aimed to explore the effects of sufentanil on the malignant behavior of lung cancer cells. H460 and H1299 lung cancer cell lines were selected for in vitro experiments. The MTT assay was conducted to detect cell viability. Proliferation ability was determined by colony formation and EdU assays. Transwell assays were performed to measure migration and invasion abilities. Western blotting was used to detect the expression of related proteins. LiCl was used to activate the Wnt/β-catenin signaling pathway. Sufentanil decreased the proliferation, migration, and invasion of H460 and H1299 cells. The protein expression levels of vimentin, N-cadherin, β-catenin, c-Myc, and MMP2 were downregulated, while those of E-cadherin and ZO-1 were upregulated after sufentanil treatment. LiCl treatment reversed the effects of sufentanil on H460 and H1299 cells. Sufentanil inhibited the proliferation, migration, invasion, and epithelial–mesenchymal transition of lung cancer cells by regulating the Wnt/β-catenin signaling pathway.

## Graphical abstract

**Figure:**
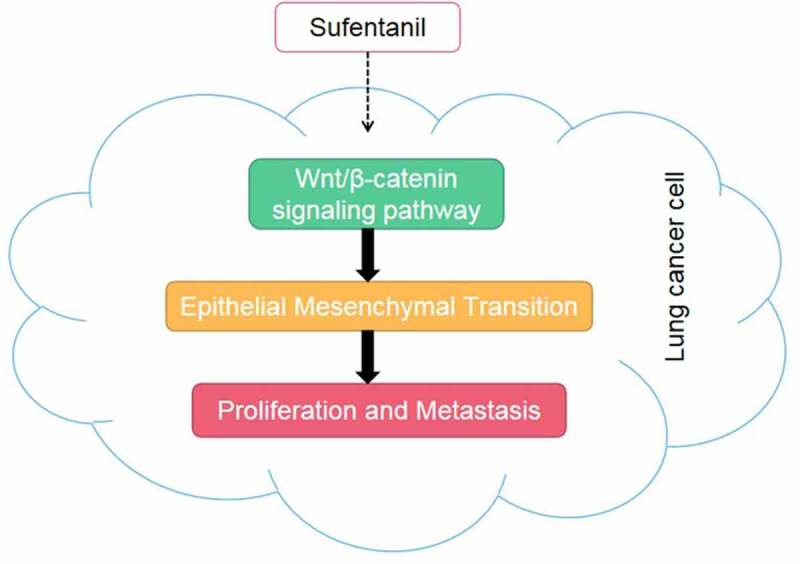

## Highlights

- Sufentanil decreased the viability of lung cancer cells.
- Sufentanil decreased proliferation, migration, and invasion of lung cancer cells.
- Sufentanil inhibited the Wnt/β-catenin signaling pathway.

## Introduction

Lung cancer is the most common malignancy and the leading cause of cancer-related death, accounting for 17% of new cancer cases worldwide. The incidence and mortality rate of lung cancer in China ranks first among the various cancers [1,2]. Lung cancer is divided into small cell lung cancer and non-small cell lung cancer, with non-small cell lung cancer accounting for 85% of cases [3]. Currently, the treatment of lung cancer is mainly based on radiotherapy, chemotherapy, and surgery [4]. In recent years, targeted drugs, such as gefitinib, for epidermal growth factor receptor (EGFR) mutations in lung cancer have gradually entered the clinic [5]. However, the cost of targeted drugs is high, and there is drug resistance in the later stages, which also drives the development of new drugs.

It is generally believed that epithelial–mesenchymal transition (EMT) is closely related to the invasion and migration of lung cancer cells [6,7]. EMT is a phenomenon in which epithelial cells transform into mesenchymal cells after stimulation by extracellular factors [8]. Once EMT is activated, adhesion between tumor cells is lost, allowing tumor cells to gain the ability to migrate and invade, and metastasize to remote organs or tissues [9]. EMT is a complex multi-step procedural process involving the regulation of multiple genes, including the upregulation of mesenchymal genes such as N-cadherin or vimentin, and the downregulation of epithelial genes such as E-cadherin or ZO-1 [10]. Therefore, inhibiting EMT is undoubtedly an important strategy for the treatment of tumor metastasis.

Sufentanil, which mainly acts on the opioid μ receptor, is a commonly used opioid anesthetic in the clinic. The analgesic effect of sufentanil is 5–10 times better than that of fentanyl [11]. Sufentanil is a fat-soluble substance with a high binding rate to human plasma proteins [12]. It has been found that sufentanil preconditioning has a protective effect against myocardial ischemia-reperfusion [13]. In recent years, many studies have confirmed that sufentanil has antitumor effects. Wu et al. [14] found that sufentanil inhibited the proliferation and induced apoptosis of gastric cancer cells in vitro. Although anesthetics may suppress tumor growth and metastasis, the underlying molecular mechanisms remain unclear.

This study is the first time to apply sufentanil to the treatment of lung cancer. Therefore, we investigated the effects of sufentanil on proliferation, migration, and invasion of H460 and H1229 lung cancer cells. We hypothesized that sufentanil suppresses the growth and metastasis of H460 and H1229 cells by inhibiting EMT development and the Wnt/β-catenin signaling pathway.

## Materials and methods

### Cell culture and treatment

The human lung cancer cell lines H460 and H1299 were provided by the Cell Bank of the Chinese Academy of Sciences (Shanghai, China). The cells were cultured in DMEM (Gibco, CA, USA) containing 10% fetal bovine serum (Gibco), 100 U/mL penicillin, and 100 U/mL streptomycin at 37°C and 5% CO_2_. The cells were then randomly divided into a control group, sufentanil group (0.5, 1, 2 nM), and sufentanil+LiCl group (2 nM sufentanil+10 mM LiCl) [15], and cultured for 24 h.

### Determination of cell viability

Cells from each group (5 × 103 cells/well) were seeded into a 96-well plate [16]. Then, 20 μL MTT solution (Beyotime Institute of Biotechnology, Shanghai, China) was added and the cells were cultured for 4 h. Next, the supernatant was collected and 150 μL DMSO was added. The absorbance (A) was measured at 490 nm using a microplate reader (BioTek, VT, USA). The cell viability was determined relative to the OD values of the control group.

### Transwell assays

Cell migration and invasion were measured using transwell assays [17]. The lower chamber contained 400 μL medium containing 10% fetal calf serum, and 200 μL cell suspension was added to the upper chamber (Matrigel was used for invasion determination and migration determination without Matrigel). The cells were then cultured for 24 h. The cells at the bottom of the transwell chamber were fixed with 4% paraformaldehyde and stained with 0.5% crystal violet. Finally, the microscope was used to observe the migrated or invaded cells.

### Colony formation assay

The cells were seeded in a 6-well plate, and 2 mL of 0.6% agarose was added [18]. After the agarose solidified, the cells were suspended in 0.3% agarose at 37°C. The cell suspension was then placed on solidified agarose at a density of 2 × 10^3^ cells/well and incubated at 4°C for 10 min. The cells were then cultured at 37°C for 14 days. Finally, the number of clones formed was counted under a microscope.

### EdU assay

The EdU assay was used to detect cell proliferation [19]. Cells were seeded in 96-well plates. According to the EdU kit instructions, cells were incubated with EdU labeling for 2 h and then fixed with 4% paraformaldehyde. After glycine decolorization, 0.5% Triton was added for 10 min to permeate the cells. The cells were then washed with PBS and stained with Apollo staining solution. Finally, EdU-positive cells were captured using a fluorescence microscope.

### Western blot

Cells were treated with RIPA lysis buffer to obtain proteins [20]. Protein concentration was measured using a BCA kit (Beyotime, Nantong, Jiangsu, China). Proteins were separated using sodium dodecyl sulfate-polyacrylamide gel electrophoresis and then transferred to a PDF membrane (Sigma, St Louis, MO, USA). Membranes were incubated with primary antibodies against E-cadherin (1:800; Abcam, Cambridge, UK), vimentin (1:1000; Abcam), N-cadherin (1:1500; Abcam), ZO-1 (1:600; Abcam), β-catenin (1:2000; Abcam), c-Myc (1:1200; Abcam), MMP2 (1:1000; Abcam), and GAPDH (1:3000; Abcam) at 4°C overnight. The next day, membranes were incubated with a secondary antibody against rabbit IgG (1:1000; Abcam) for 2 h. The bands were analyzed using an enhanced chemiluminescence system (Super Signal West Pico Substrate, Thermo Fisher Scientific), and ImageJ software (National Institutes of Health, Bethesda, MD, USA) was used for quantification.

## Statistical analysis

The data were statistically analyzed using GraphPad Prism 8 software and expressed as mean ± standard deviation. The difference between the two groups was compared using Student’s t-test, and comparisons among multiple groups were performed using one-way analysis of variance (ANOVA). Statistical significance was set at P < 0.05.

## Results

This study demonstrated that sufentanil suppressed the proliferation, migration, and invasion of lung cancer cells by inhibiting EMT and the Wnt/β-catenin signaling pathway. LiCI treatment reversed the effects of sufentanil on lung cancer cells.

### Sufentanil decreased the viability of lung cancer cells

**Figure 1.**
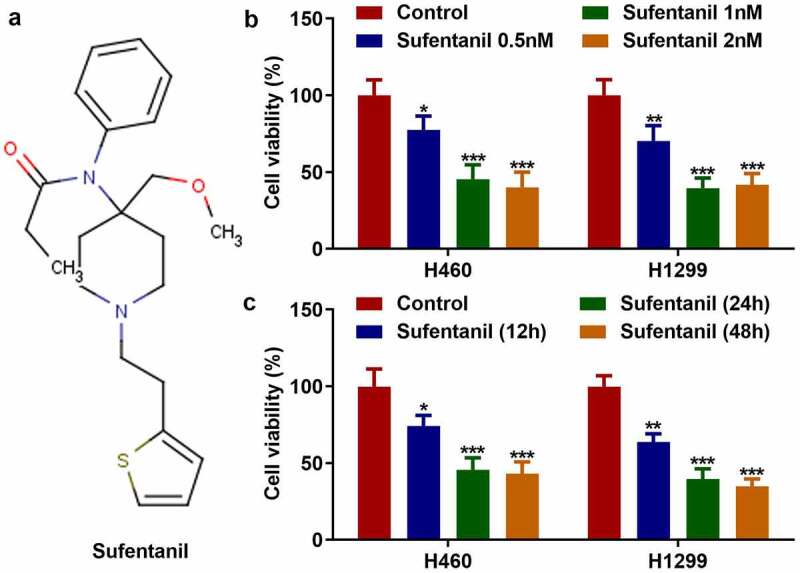
Sufentanil decreased the viability of lung cancer cells. (a) The molecular formula of sufentanil. (b) The cell viability of the H460 and H1299 was detected by MTT assay after sufentanil (0.5, 1, 2 nM) treatment. (c) The cell viability of the H460 and H1299 was detected by MTT assay at 12, 24, 48 h, after 2 nM sufentanil treatment.

The molecular formula of sufentanil is shown in Figure 1a. We found that sufentanil decreased the viability of H460 and H1299 cells in a dose-dependent manner (Figure 1b); 2 nM sufentanil was selected for further experiments. In addition, 2 nM sufentanil decreased the viability of H460 and H1299 cells in a time-dependent manner (Figure 1c). Therefore, we selected 2 nM sufentanil for interaction with lung cells for 48 h as the condition for the follow-up experiment.

*P < 0.05, **P < 0.01, ***P < 0.001 VS Control group.

## *Sufentanil decreased the proliferation, migration, and invasion abilities of the* lung *cancer cells*

**Figure 2.**
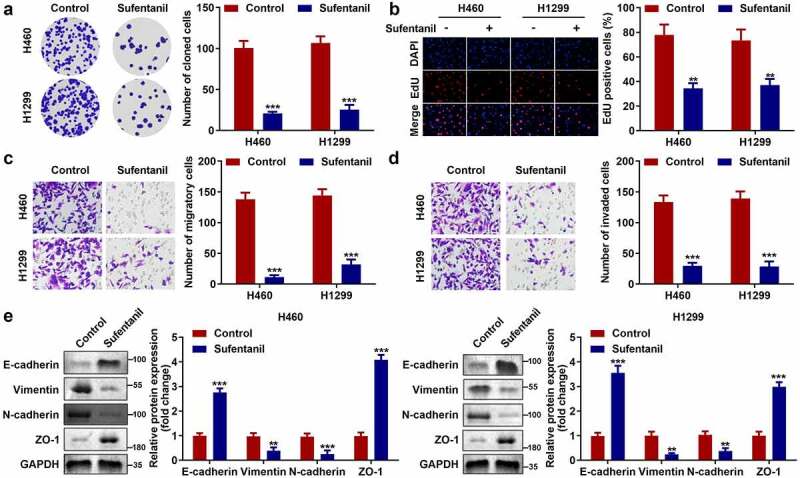
Sufentanil decreased the proliferation, migration, and invasion abilities of the lung cancer cells. (a-b) The cell proliferation of the H460 and H1299 cells was measured by colony formation and EdU assays after 2 nM sufentanil treatment. (c-d) The migration and invasion abilities of the H460 and H1299 cells were tested by transwell assay after 2 nM sufentanil treatment. (e) The protein expressions of Vimentin, N-cadherin, E-cadherin and ZO-1 were measured by western blot after 2 nM sufentanil treatment.

Next, we explored the effects of sufentanil on the growth and metastasis of H460 and H1299 cells. The colony formation assay showed that sufentanil significantly decreased the number of cloned cells (Figure 2a), which was consistent with the results of the EdU assay (Figure 2b). Furthermore, we found that sufentanil dramatically decreased cell migration (Figure 2c) and invasion (Figure 2d) cells. In addition, we found that sufentanil dramatically downregulated vimentin and N-cadherin protein expression, and upregulated E-cadherin and ZO-1 protein expression in H460 and H1299 cells (Figure 2e).

*P < 0.05, **P < 0.01 VS Control group.

### Sufentanil inhibited the Wnt/β-catenin signaling pathway

**Figure 3.**
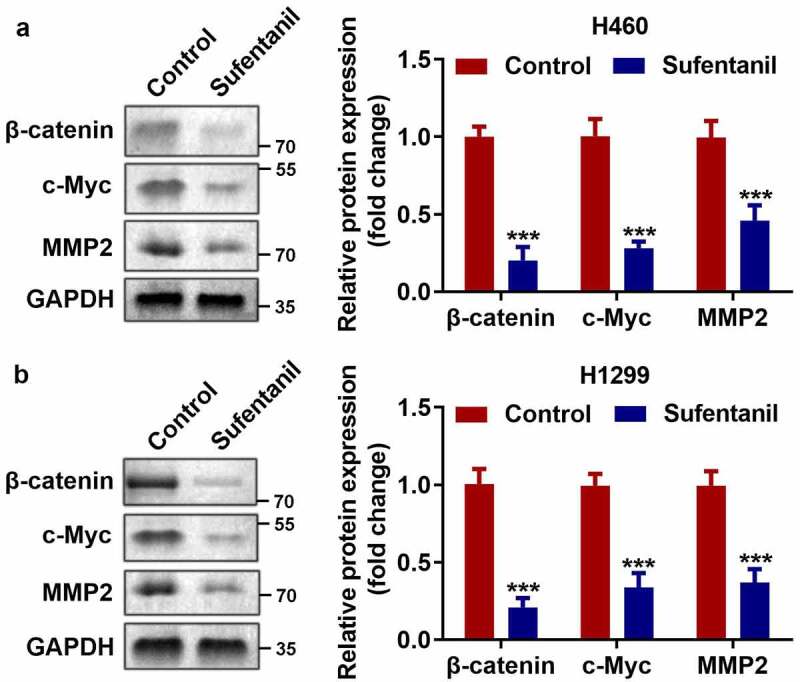
Sufentanil inhibited the Wnt/β-catenin signaling pathway. (a-b) The protein expressions of β-catenin, c-Myc, and MMP2 of the H460 and H1299 cells were measured by western blot after 2 nM sufentanil treatment.

As shown in Figure 3a-b, we found that sufentanil dramatically downregulated the protein expression of β-catenin, c-Myc, and MMP2 in H460 and H1299 cells.

**P < 0.01 VS Control group.

### LiCl activated the Wnt/β-catenin signaling pathway

**Figure 4.**
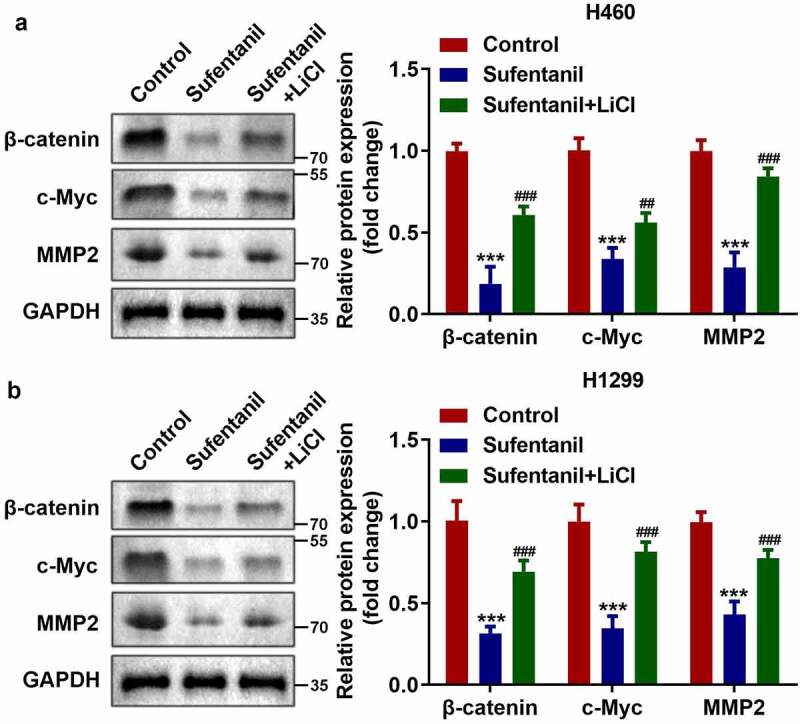
LiCl activated the Wnt/β-catenin signaling pathway. (a-b) The protein expressions of β-catenin, c-Myc, and MMP2 of the H460 and H1299 cells were measured by western blot after 2 nM sufentanil and 10 mM LiCl treatment.

Subsequently, to explore the specific mechanism of Wnt/β-catenin signaling pathway in lung cancer cells, we used LiCI (Wnt activator) to activate the Wnt/β-catenin signaling pathway. As shown in Figure 4a-b, the protein expression of β-catenin, c-Myc, and MMP2 in H460 and H1299 cells was significantly upregulated after LiCI treatment.

### Activation of the Wnt/β-catenin signaling pathway reversed the sufentanil effects

**Figure 5.**
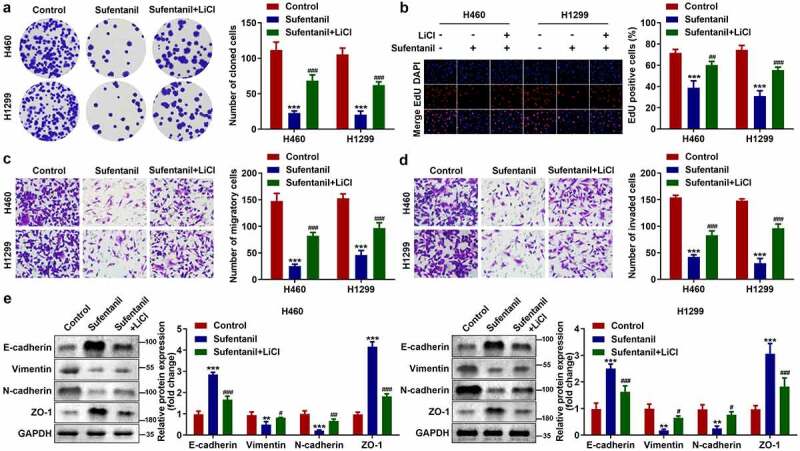
Activation of the Wnt/β-catenin signaling pathway reversed the sufentanil effects. (a-b) The cell proliferation of the H460 and H1299 cells was measured by colony formation and EdU assays after 2 nM sufentanil and 10 mM LiCl treatment. (c-d) The migration and invasion abilities of the H460 and H1299 cells were tested by transwell assay after 2 nM sufentanil and 10 mM LiCl treatment. € The protein expressions of Vimentin, N-cadherin, E-cadherin and ZO-1 were measured by western blot after 2 nM sufentanil treatment.

Finally, we found that LiCI treatment reversed the effects of sufentanil on the number of cloned (Figure 5a), EdU-positive (Figure 5b), migrated (Figure 5c), and invaded (Figure 5c) H460 and H1299 cells. Additionally, the protein expression of vimentin and N-cadherin was upregulated, and that of E-cadherin and ZO-1 was downregulated by LiCI (Figure 5e).

## Discussion

In this study, sufentanil suppressed the proliferation, migration, and invasion of lung cancer cells by inhibiting EMT and the Wnt/β-catenin signaling pathway. LiCI treatment reversed the effects of sufentanil on lung cancer cells.

Various anesthetics exhibit potent anti-tumor effects. For example, Yang et al. [21] confirmed that lidocaine inhibits cell growth and metastasis, and promotes apoptosis in lung cancer cells. Li et al. [22] found that a high dose of anesthetic dramatically suppressed the growth of breast cancer cells. In recent years, sufentanil has been used to treat various cancers. Zhao et al. [23] demonstrated that sufentanil improved coagulation and immune function following colon cancer resection. Tang et al. [24] also confirmed that sufentanil relieves inflammatory responses after esophageal cancer surgery. However, to the best of our knowledge, the use of sufentanil for lung cancer is limited. In the present study, we demonstrated that sufentanil significantly suppressed proliferation and induced apoptosis of H460 and H1299 cells. These results preliminarily indicate that sufentanil has an antitumor effect on lung cancer.

Distant metastasis is the main barrier to lung cancer treatment [25,26]. Augmenting evidence has demonstrated that EMT plays an important role in the invasion and metastasis of lung cancer cells [27,28]. The EMT of tumors is the process of transforming epithelial cells with polarity of tumor cells into mesenchymal cells with migration ability to obtain stronger invasion and migration ability [29]. In addition to changes in tumor cell morphology, the process of EMT is also accompanied by changes in related molecular markers, such as the downregulation and expression of epithelial cell markers such as E-cadherin or ZO-1, and upregulation of the expression of mesenchymal cell phenotypic markers such as vimentin and N-cadherin [30]. Accumulating evidence has demonstrated that the inhibition of EMT can relieve the progression of lung cancer. Li et al. [31] showed that esculetin regulates EMT in A549 cells by downregulating the expression of vimentin and Snail and upregulating the expression of E-cadherin, eventually inhibiting cell growth. Wang et al. [32] confirmed that the expression of E-cadherin was upregulated, and that of N-cadherin, vimentin, and fibronectin was downregulated after icotinib treatment. In this study, we found that sufentanil significantly inhibited EMT in lung cancer cells, manifested by the acquisition of mesenchymal features and an increase in the migration and invasion ability of lung cancer cells. These results indicate that sufentanil inhibits the development of lung cancer by suppressing EMT.

The Wnt/β-catenin signal transduction pathway plays an important role in the development of many tissues, such as the lungs, kidneys, and nerves. Additionally, it is a key regulator of EMT [33,34]. The classic Wnt signaling pathway prevents β-catenin degradation in the cytoplasm by inhibiting GSK3β-mediated phosphorylation. A large amount of accumulated β-catenin then enters the nucleus and binds to the downstream promoter TCF/LCF3 complex to regulate downstream target genes, thus inducing the initiation of the EMT program [35,36]. Moreover, the Wnt signaling pathway activates MMP family related genes through classical and non-classical pathways, which eventually reduces the expression of E-cadherin and increases the expression of vimentin, thus promoting the process of EMT [37]. In this study, the protein expression levels of β-catenin, c-Myc, and MMP2 were downregulated after sufentanil treatment, indicating that sufentanil inhibits the Wnt/β-catenin signaling pathway. However, LiCl, an activator of Wnt/β-catenin signaling, reversed the effects of sufentanil on the proliferation and EMT of lung cancer cells. These results imply that sufentanil may inhibit EMT development by regulating the Wnt/β-catenin signaling pathway, eventually relieving the growth and metastasis of lung cancer cells.

## Conclusion

In summary, this study is the first to investigate the role of sufentanil in lung cancer. Our results showed that sufentanil suppressed the proliferation and EMT of H460 and H1299 cells by inhibiting the Wnt/β-catenin signaling pathway. Therefore, sufentanil may be a promising therapeutic agent for lung cancer.

## Data Availability

The datasets used and analyzed during the current study are available from the corresponding author on reasonable request.
